# Strigolactones Promote Leaf Elongation in Tall Fescue through Upregulation of Cell Cycle Genes and Downregulation of Auxin Transport Genes in Tall Fescue under Different Temperature Regimes

**DOI:** 10.3390/ijms20081836

**Published:** 2019-04-13

**Authors:** Qiannan Hu, Shuoxin Zhang, Bingru Huang

**Affiliations:** 1 College of Forestry, Northwest A&F University, Yangling, Xianyang 712100, Shaanxi, China; qnanhu@gmail.com; 2 Department of Plant Biology and Pathology, Rutgers, the State University of New Jersey, New Brunswick, NJ 08901, USA

**Keywords:** strigolactones, auxin, leaf elongation, heat stress, grass

## Abstract

Strigolactones (SLs) have recently been shown to play roles in modulating plant architecture and improving plant tolerance to multiple stresses, but the underlying mechanisms for SLs regulating leaf elongation and the influence by air temperature are still unknown. This study aimed to investigate the effects of SLs on leaf elongation in tall fescue (*Festuca arundinacea*, cv. ‘Kentucky-31’) under different temperature regimes, and to determine the interactions of SLs and auxin in the regulation of leaf growth. Tall fescue plants were treated with GR24 (synthetic analog of SLs), naphthaleneacetic acid (NAA, synthetic analog), or N-1-naphthylphthalamic acid (NPA, auxin transport inhibitor) (individually and combined) under normal temperature (22/18 °C) and high-temperature conditions (35/30 °C) in controlled-environment growth chambers. Exogenous application of GR24 stimulated leaf elongation and mitigated the heat inhibition of leaf growth in tall fescue. GR24-induced leaf elongation was associated with an increase in cell numbers, upregulated expression of cell-cycle-related genes, and downregulated expression of auxin transport-related genes in elongating leaves. The results suggest that SLs enhance leaf elongation by stimulating cell division and interference with auxin transport in tall fescue.

## 1. Introduction

Leaf elongation is a decisive factor of shoot biomass production in leafy crops, including perennial grasses that are used as forage [1]. Two developmental processes that control leaf elongation are cell elongation or expansion and rate of cell division [2,3]. In grass species, active cell division and expansion occur in the leaf meristematic zone, which is located at the base of an elongating leaf [4,5]. Factors regulating either cell number or cell length may affect the overall leaf length and elongation rate. 

Classic phytohormones, including auxin, gibberellic acid (GA), and cytokinins, play key roles in controlling cell division and cell expansion, with a hormone-specific mode of action for each of them [6,7,8]. Recently, SLs have also been named as plant hormones that play important roles in regulating plant architecture, such as shoot branching [9,10]. Exogenous application of a bioactive, synthetic SL, *rac*-GR24((3aR*,8bS*,E)-3-(((R*)-4-methyl-5-oxo-2,5-dihydrofuran-2-yloxy)methylene)-3,3a,4,8b-tetrahydro-2H-indeno[1,2-b]furan-2-one) results in shortening of the mesocotyl [11] and hypocotyl [12], and stimulating elongation of internodes [13,14], mesocotyls [11,15], and roots [16,17,18,19] through the regulation of cell division. In dicotyledonous plants, such as *Arabidopsis* (*Arabidopsis thaliana*), SLs have also been reported to modify leaf shape [20,21,22]. In addition, other studies reported that leaf blade [20,23] and petiole length [20,21] in SL-deficient mutants were reduced in comparison to wild-type *Arabidopsis*. 

Auxin is known to play an important role in leaf growth and elongation [24]. Some studies report that increased auxin levels have negative effects on leaf growth. It has been shown that transgenic, auxin-overproducing petunia (*Petunia hybrida*) had smaller and narrower leaves than the wild type [25]. Similarly, *Arabidopsis* auxin-overproducing mutants, *sur1* and *sur2*, showed reduced leaf area [26]. Moreover, auxin content normally decreases during leaf growth [27], which suggests that auxin plays an inhibitory role in leaf development and the decreasing auxin level might be a precondition for full leaf expansion. Auxin and SLs interact in regulating shoot development [28]; for example, SLs interfere with auxin transport in both *Arabidopsis* and rice (*Oryza sativa*) [9,29,30]. Additionally, SLs act downstream of auxin to modulate the shoot architecture in peas (*Pisum sativum*) and *Arabidopsis* [31]. Hayward and Leyser [28] have further suggested that auxin and SLs affect each other’s endogenous levels and distribution within plants, which have direct implications on plant architecture. It has, however, not been well documented whether SLs affect leaf elongation through crosstalk with auxin in leaves of monocotyledons, including perennial grasses.

Leaf elongation is sensitive to environmental factors and is inhibited by abiotic stresses, including heat stress [32]. The optimal temperature for leaf development in cool-season grasses is 18–24 °C, but air temperature in the summer often exceeds this optimal temperature range, thereby restricting leaf growth [33,34]. A few studies have reported that SLs may mediate plant response to abiotic stresses. Ha et al. [35] suggest that SLs induce stomatal closure, thereby improving drought tolerance, and Zhuang et al. [36] propose that SLs participate in increasing drought tolerance in tall fescue through the regulation of tiller production. Although plant responses to SLs have been studied in a number of cases [37], the positive regulatory role of SLs in heat stress has been rarely discussed. Current research has shown that the germination rates of seeds in both *Arabidopsis* WT and SLs-deficient *max1* plants were increased by GR24 treatment under heat stress, but GR24 had no effect on germination rate in mutant SLs-signaling *max2* plants [38]. Moreover, the expression level of light harvesting-associated genes and chlorophyll content were enhanced upon GR24 treatment in SLs-deficient tomato mutants (*Sl-ORT1*) and WT plants under light stress [39]. These studies serve as indirect evidence, suggesting SLs’ positive effects on heat stress tolerance, as light and heat stresses cause similar injury to the structure of thylakoids and PSII complexes [40]. Marzec and Muszynska [41] report that heat stress upregulated the expression of *MAX3*, the SLs-synthesis gene in *Arabidopsis*, which again indicates that SLs play a role in the plant tolerance to heat stress. Recently, Hu et al. [42] reported that GR24 stimulates crown root elongation under heat stress in tall fescue.

We hypothesize that SLs affect the cell divisions and/or expansion that ultimately control leaf elongation, thereby alleviating the heat inhibition of leaf elongation, and that these SLs effects involve an interaction with auxin. This study aimed to investigate the effects of SLs on leaf growth in tall fescue growing under different temperatures and to determine the interaction between SLs and auxin regulating leaf elongation.

## 2. Results

### 2.1. Effects of GR24 and Auxin on Leaf Elongation in Tall Fescue under Different Temperature Regimes

**Figure 1:**
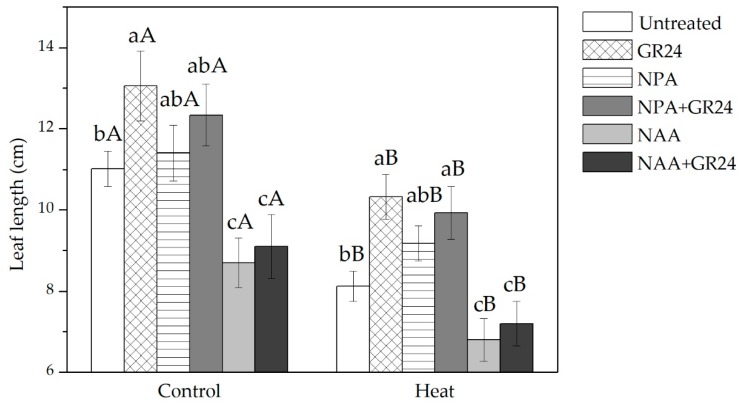
Leaf length of plants under each treatment. Lowercase letters are for comparison between different chemical treatments, and uppercase letters are for comparison between treatments under two temperature conditions.

GR24 treatment resulted in an increase of 18% in leaf length in tall fescue growing under normal temperature (Figure 1). Heat stress severely suppressed leaf length by 26%, from 11.0 cm in the normal temperature control plants to 8.1 cm in the heat-stressed plants (Figure 1). GR24 treatment mitigated the inhibitory effect of heat stress on leaf elongation, resulting in a 27% increase in leaf length compared to untreated plants under heat stress (Figure 1).

Treatment with the auxin transport inhibitor NPA had no significant effect on leaf elongation under either temperature condition, while the combined treatment of GR24 and NPA exhibited a positive effect under heat stress (Figure 1). NAA inhibited leaf elongation and GR24 did not alleviate the adverse effect of NAA (Figure 1).

**Figure 2:**
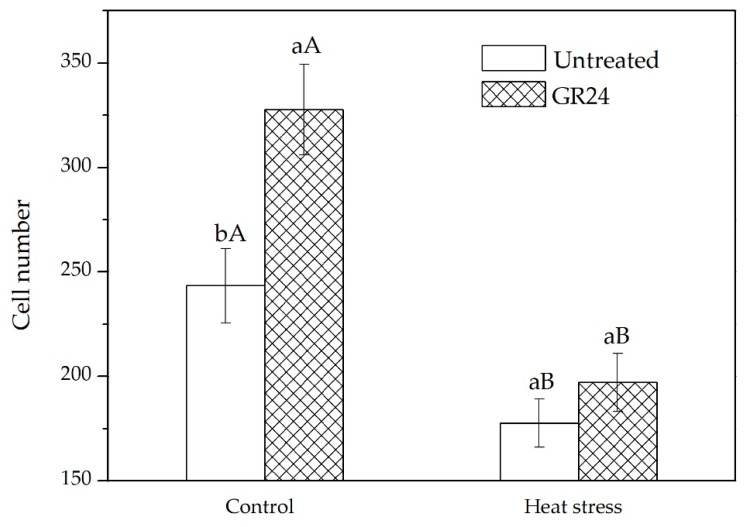
Effects of GR24 on number of leaf cells in the meristematic zone under two different temperature regimes. Each value represents a mean of six to eight leaves for each seedling. Error bars indicate standard deviation. Lowercase letters are for comparison between GR24-treated and untreated plants, and uppercase letters are for comparison between treatments under two different temperature regimes.

Leaf anatomical features of GR24-treated and untreated seedlings were examined to determine whether the GR24 treatment affects leaf cell length and number. There was no significant difference in cell length between GR24-treated and untreated seedlings (data not shown), while the GR24-treated plants had a higher cell number in the meristematic zone than in untreated plants (Figure 2).

### 2.2. Expression of Genes Related to Cell Cycle, Auxin Transport, and SLs Signaling in Leaves

**Figure 3:**
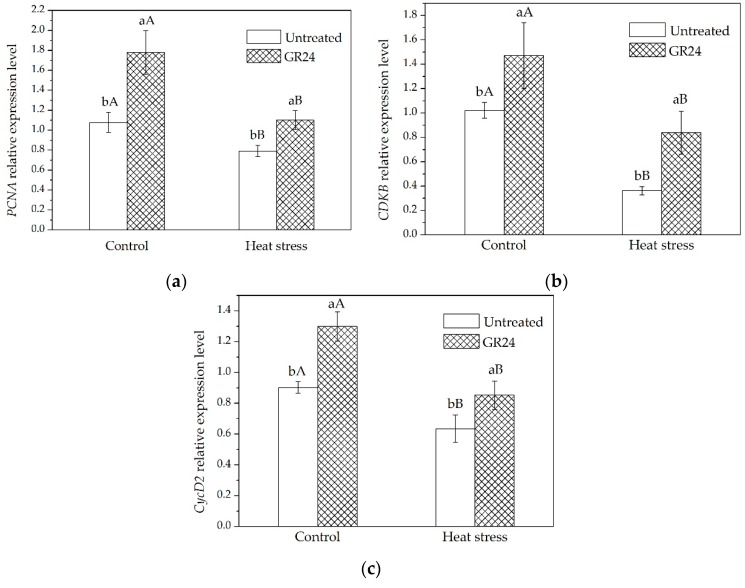
(**a**) *PCNA* expression level in leaves treated with or without GR24 under two different temperature regimes; (**b**) *CDKB* expression level in leaves treated with or without GR24 under two different temperature regimes; (**c**) *CycD2* expression level in leaves treated with or without GR24 under two different temperature regimes. Lowercase letters are for comparison between different chemical treatments, and uppercase letters are for comparison between treatments under two temperature conditions.

Expression of several cell-cycle-related genes, including *Proliferating Cell Nuclear Antigen* (*PCNA*), *Cyclin-D2* (*CycD2*) and *Cyclin-Dependent Kinase B* (*CDKB*), were measured using q-PCR analysis in order to investigate whether the positive regulation of cell number in leaves by GR24 is associated with changes in cell division. Figure 3 shows that the expression of those genes was upregulated upon GR24 treatment under both temperature conditions.

**Figure 4:**
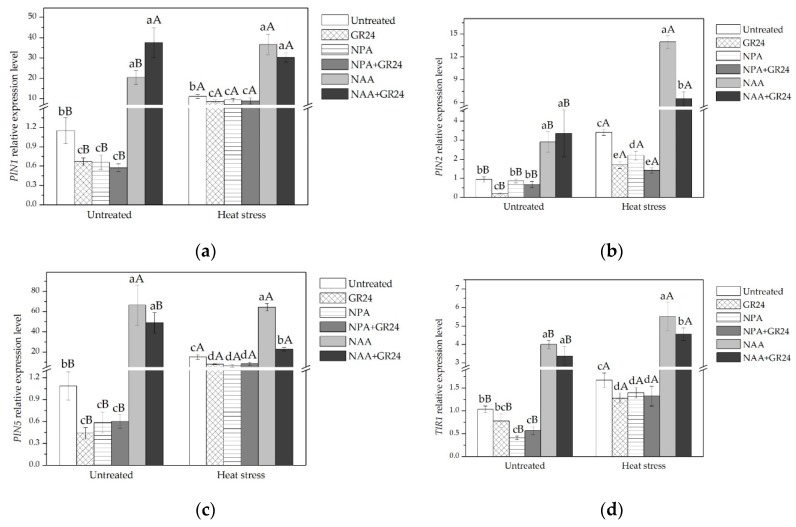
(**a**) *TIR1* expression level in leaves for all treatments; (**b**) *PIN2* expression level in leaves for all treatments; (**c**) *PIN5* expression level in leaves for all treatments; (**d**) *PIN1* expression level in leaves for all treatments. Lowercase letters are for comparison between different chemical treatments, and uppercase letters are for comparison between treatments under two temperature conditions.

The expression levels of three auxin transport genes, *PIN1*, *PIN2*, and *PIN5*, and the auxin receptor gene, *TIR1*, are measured in leaves under all treatments to determine whether GR24 promotes leaf elongation by interfering with auxin transport or perception. Data show that the expression levels of all three auxin transport-related genes decreased in leaves upon GR24 treatment, but increased significantly in leaves treated with NAA under both temperature conditions (Figure 4). The expression levels of *PIN1* and *PIN5* decreased in leaves upon NPA treatment. High temperature induced the expression of three auxin transport-related genes, especially the expression levels of *PIN1* and *PIN5*. GR24 treatment led *PIN2* expression to be more significantly inhibited than the NPA treatment, while the expression levels of other auxin transport genes do not show any significant differences for either of the two treatments under normal temperature condition. The expression levels of *PIN1*, *PIN5*, and *TIR1* did not differ significantly among GR24, NPA, and NPA + GR24 treatments in plants exposed to high temperature.

**Figure 5:**
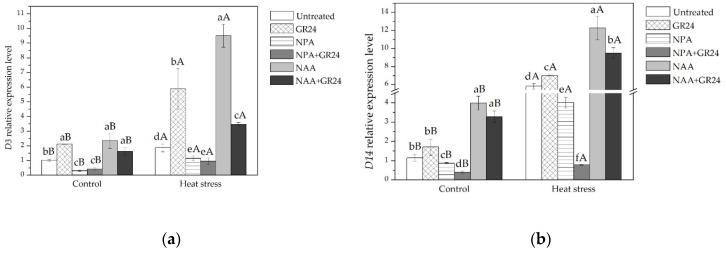
(**a**) *D3* expression level in leaves for all treatments; (**b**) *D14* expression level in leaves for all treatments. Lowercase letters are for comparison between different chemical treatments, and uppercase letters are for comparison between treatments under two temperature conditions.

D3 and D14 family proteins are involved in SLs perception. *D3* and *D14* expression levels were analyzed to determine changes in SLs signaling genes in responses to GR24, NAA or NPA. The expression of these two genes increased significantly following NAA and NAA + GR24 treatment while they were reduced upon NPA and NPA + GR24 treatment under both temperature conditions (Figure 5). *D3* expression level could be induced upon GR24 treatment under both temperature conditions while *D14* expression level could only be induced upon GR24 treatment at high temperatures. The *D3* gene expression level following treatment with GR24 or NAA under heat stress conditions was much higher than that of plants under normal temperature conditions (Figure 5).

## 3. Discussion

The biological functions of SLs are still largely unknown, although the effects of SLs on shoot branching and leaf shape have been previously reported [22,23]. This study first found that SLs were able to mitigate the inhibitory effects of high temperature on leaf elongation in tall fescue. It is well known that leaf elongation is governed by both cell division and expansion; however, whether SLs-stimulated leaf elongation is due to effects on either or both of these developmental processes is not well documented. Our results demonstrate that growth-promoting effects of SLs on leaf growth in tall fescue are mainly due to the increased number of cells in individual leaves, presumably by maintaining active cell division even at high temperatures (Figure 2). Genes related to cell cycle activities are closely associated with cell division [43,44,45]. *PCNA* (*Proliferating Cell Nuclear Antigen*) is an important gene involved in DNA replication and cell cycle regulation [46]. *CycD2* (*Cyclin-D2*) and *CDKB* (*Cyclin-Dependent Kinase B*) play roles in promoting activities of the cell cycle [47,48]. These three genes are associated with different stages of the cell cycle. In our study, the expression levels of *PCNA*, *CycD2* and *CDKB* are increased upon GR24 treatment (Figure 3). Sun et al. [18] report that treating plants with GR24 upregulate the expression of the cell cycle activity-related gene, *CYCB1;1*, in rice. These results indicate that the number of leaf cells increases by SLs may have resulted from up regulation of the genes that control cell division in the leaves of tall fescue.

Previous studies have exhibited that SLs interact with auxin, controlling leaf shape in alfalfa [22] and shoot branching in *Arabidopsis* [28,29,49,50]. Our study demonstrated that SLs interacted with auxin in regulating leaf growth in tall fescue. In contrast with the positive effects of SLs on leaf elongation in tall fescue, the NAA treatment suppressed leaf elongation in our study (Figure 1), consistent with previous studies regarding other plant species [51,52,53,54]. A higher level of auxin in *Arabidopsis* has been reported to inhibit leaf growth [52]. Borah and Baruah [51] report that rice treated with IAA has a lower leaf biomass than untreated controls. Mulkey et al. [53] report that 1 µM IAA strongly inhibits root growth in maize (*Zea mays*). SLs synthesis and signaling pathways may control shoot architecture or branching by regulating auxin transport [28,50,55,56]. Chemicals that act on polar auxin transport are useful for studying the relationship between GR24 and auxin transport. NPA is a widely used inhibitor of auxin transport [57,58], while GR24 and NPA are shown to have similar effects on bud and root elongation [42,59]. In order to determine whether SLs could reverse the adverse effects of auxin or inhibit auxin transport, GR24 is applied to plants treated with NAA or NPA in our study. Our results indicated that the GR24 + NAA treatment did not improve leaf elongation under two different temperature conditions, which suggested that SLs were not able to directly reverse the negative effects of NAA on leaf elongation in tall fescue. On the contrary, the NPA and NPA + GR24 treatments improved leaf elongation under heat stress, similar to the GR24 treatment. However, the hidden mechanisms explaining how SLs affect auxin transport, resulting in the elongation of leaves, deserve further study.

Auxin efflux carriers such as PIN-FORMED (PIN) family proteins play key roles in auxin distribution [60], which has a significant influence on the regulation of cell division [61]. Lazar et al. [62] report that the auxin influx gene, *AUX1*-like, and auxin efflux genes *PIN1*, *PIN3*, and *PIN4* are overexpression in SLs mutants of *Arabidopsis*. Saini et al. [54] showed that the PIN1 protein overaccumulates in *max* mutants. Hao et al. [63] report that *PIN2* and *PIN5* are induced by heat stress. *Transport Inhibitor Response1* (*TIR1*) is an auxin receptor associated genes [64,65]. It has been reported that the auxin receptor mutant *tir1-1* shows reduced sensitivity to GR24 relative to the WT [66]. Mayzlish-Gati et al. [67] show that SLs’ regulation of a plant’s response to low Pi conditions is correlated with increased *TIR1* expression. In the present study, the expression levels of three auxin efflux carrier genes (*PIN1*, *PIN2*, and *PIN5*) and *TIR1* were measured to determine whether SLs stimulate leaf elongation through effects on auxin transport or perception. Our results showed that the expression of *PIN1*, *PIN2*, *PIN5*, and *TIR1* were suppressed by GR24 application under both temperature conditions, with similar results being seen for NPA (Figure 4). For *PIN1*, *PIN2*, and *PIN5*, suppression was more significant under high temperature. While the expression of *PIN1* and *PIN5* was significantly upregulated upon NAA and NAA + GR24 treatments under both temperature conditions.

Auxin may additionally affect SLs signaling in the regulation of leaf elongation. The *D3* gene encodes an F-box protein, and the *D14* gene encodes a protein that belongs to the α/β-fold hydrolase family, and both are SL receptors [68,69,70]. It has been found that NAA treatment results in the increased expression of the two SLs-signaling genes, *D3* and *D14*, while NPA downregulates the expression of *D3* and *D14* (Figure 5). Similarly, one study found that SLs response was upregulated by auxin via the *D3* gene component of SLs signaling during shoot branch formation in *Arabidopsis* [29]. In addition, it has been reported that the transcription of *CCD7* and *CCD8*, two SLs biosynthesis genes is upregulated by auxin [28,71]. While the interactions between auxin and SLs in the regulation of shoot development are widely studied [28], the manners in which these two hormones regulate leaf elongation need further research.

In summary, our study demonstrated that SLs increased leaf length and mitigated the adverse effect of heat stress on leaf elongation in tall fescue by promoting cell division in the leaf meristematic zone. SLs could affect auxin transport, and both SLs and auxin might regulate leaf elongation in grass species by mutually acting on one another; however, the underlying mechanisms associated with how they interact are worth further study. This research offers a new insight regarding the breeding of new varieties of leafy crops with high biomass production, particularly for enhancing leaf growth in environments with elevated temperatures as a primary abiotic stress.

## 4. Materials and Methods

### 4.1. Plant Materials and Growth Conditions

Seeds of tall fescue (cv. ‘Kentucky-31’) were planted in fritted clay medium (Profile Products, Deerfield, IL) on 1 May 2017. After three weeks, uniformly sized seedlings were rinsed with water to ensure that the roots were free of clay medium and then transplanted to a hydroponic growing medium on 22 May 2017. The bases of the seedlings’ shoots were covered by foam cubes, which were then inserted into a polystyrene board. The board was floated in a plastic box (30 × 21 × 18 cm) filled with half-strength Hoagland’s nutrient solution [72]. Forty-eight containers of plants were used in this experiment, and there were 160 plants in each container. An air pump was used to continuously inject air into the hydroponic solution to ensure appropriate aeration. The experiment was conducted in controlled-environment growth chambers (Environmental Growth Chambers, Chagrin Falls, OH, USA), maintained at a temperature of 22/18 °C (day/night), a 12-h photoperiod, 60% relative humidity, and 650 µmol m^−2^s^−1^ photosynthetically active radiation at the canopy level during seedling establishment.

### 4.2. Experimental Treatments

Before exposure to heat stress condition, three-week-old seedlings were cultured in half-strength Hoagland’s nutrient solution for 7 days. To initiate heat treatment, seedlings were exposed to 35/30 °C (day/night) for 8 days, between 31 May and 7 June 2017, while control plants were grown at 22/18 °C (day/night). Other environmental conditions were maintained as previously described.

Seedlings receiving the GR24 treatment were foliar sprayed with 0.01 µM GR24 (a synthetic analog of SLs, dissolved in acetone and diluted with water) one day prior to high-temperature exposure and every two days during the eight days of heat treatment. For NPA treatment, seedlings were foliar sprayed with 0.5 µM NPA (an auxin transport inhibitor, dissolved in dimethyl sulfoxide [DMSO] and diluted with water) 1 day prior to placement into the heat chamber and every two days during the eight days of heat treatment. For each application, 30 mL of solution were sprayed onto leaves in each container. Tween 20 was added to each treatment solution as a spreading and wetting agent. For the NAA (dissolved in 1 µM NaOH and diluted with water) treatment, plants’ root systems were first incubated overnight in half-strength Hoagland’s nutrient solution containing 1 µM NAA prior to imposition of heat stress and then transferred into half-strength Hoagland’s nutrient solution without NAA. The concentration of chemicals used in the experiment was optimized according to the pre-experiment.

Heat stress and hormone treatments were arranged in a split-plot design with heat stress treatments as main plots and hormone treatments as subplots. The normal temperature control and heat stress treatment were repeated in three growth chambers. Each hormone treatment had four replicates (four containers of plants containing 160 replicate plants), which were randomly placed in each chamber.

### 4.3. Leaf Elongation Analysis and Histological Observation

Images of leaves were acquired using a scanner (Epson EU-35, Seiko Epson Corp., Suwa, Japan) on 7 June 2017. Average leaf length was obtained using the image analysis program, Digimizer (MedCalc Software, Mariakerke, Belgium).

To examine cell number and length in individual leaves, nail polish was brushed onto the leaf surface, and a transparent negative plate of the epidermis was carefully acquired. Images were captured under a microscope (Nikon Instruments Inc., model SMZ1270, Melville, NY, USA). The number of epidermal cells in the meristematic zone of the longitudinal leaf was determined by counting the cortical cells in files extending from the ligule to the first elongated cell. Epidermal cell length was calculated by counting the cell number per unit length in the longitudinal leaf.

### 4.4. qRT-PCR Analysis

The expression level of genes associated with the cell cycle, auxin transport, and SLs signaling in leaves were analyzed via quantitative reverse transcription polymerase chain reaction (qRT-PCR). Total RNA was extracted from leaf tissue with TRIzol reagent (Life Technologies, Grand Island, NY, USA) and contaminating genomic DNA was removed with DNase (TURBO DNA-free kit; Life Technologies). Following RNA extraction, 2 µg total RNA was reverse-transcribed to cDNA using a high-capacity cDNA reverse transcription kit (Life Technologies). The synthesized cDNA was expanded in a StepOnePlus Real-Time PCR system (Life Technologies) using the following procedure: Pre-heat cycle at 95 °C for 3 min, followed by 40 cycles of 95 °C denaturation for 30 s and 60 °C annealing/extension for 60 s. Power SYBR Green PCR Master Mix (Life Technologies) was used as the intercalating dye for detecting gene expression level. Gene names and forward and reverse primer sequences are shown in Table 1. A melting curve analysis was performed for each primer pair to confirm binding specificity. The data were standardized using *Actin* as the reference gene, since its expression was constant throughout treatments. The relative expression levels between genes of interest and reference genes were calculated using the ΔΔ*C*t method.

### 4.5. Statistical Analysis

The effects of heat stress and hormone treatments and their interactions on leaf length, cell number, and gene expression levels were determined by analysis of variance with SPSS software (version 20.0, SPSS Inc., Chicago, IL, USA). Differences between mean values for each parameter were distinguished by Student’s *t*-test (α = 0.05).

## Figures and Tables

**Table 1:** Primers used in this research.

Primer Name	Forward Primer Sequence (5′–3′)	Reverse Primer Sequence (5′–3′)
*Actin*	TCTTACCGAGAGAGGTTACTCC	CCAGCTCCTGTTCATAGTCAAG
*FaPCNA*	CATCTGAGCTACCAGTGGTG	CCTCGTCCTCTTCAATCTTC
*FaCycD2*	ACTGCTCTCGGCTTGTTCAT	GAAGACTCCCTCCTCCCATC
*FaCDKB*	GTTGTTGGGAACTCCTACTG	CGATAACAGGTCAAGTCCTTC
*FaTIR1*	AGGCTGTTGGTTGGATAAAG	CCCAGCCTCCTACAGTTAT
*FaPIN1*	CTAGCTAAGTAGGACCCTAGAC	CTCAAAGCCCTAGCCTTTAC
*FaPIN2*	GGTCTAGGGATGGCTATGT	AATGTGGCGACAGACTTG
*FaPIN5*	ATGGGTTTGGCAACTACG	GGTTCATCGGCAGGTATAAG
*FaD3*	GACAGGTACTCCATCTTCCT	ATGTTCGCGAGAGCAAAG
*FaD14*	GAACGACAGCGACTACCA	ACGTAGTTCGCCGACATC
